# Association of *Rs339939442* in the *AHR* Gene with Litter Size are Inconsistent among Chinese Indigenous Pigs and Western Commercial Pigs

**DOI:** 10.3390/ani10010011

**Published:** 2019-12-19

**Authors:** Qian Zhang, Ruihua Huang, Xiang Ma, Nengjing Jiang, Wuduo Zhou, Chen Gao, Moran Zhao, Peipei Niu, Zongping Zhang, Qiang Li, Juan Zhou, Pinghua Li

**Affiliations:** 1 Institute of Swine Science, Nanjing Agricultural University, Nanjing 210095, China; zhangqian_1992@126.com (Q.Z.); rhhuang@njau.edu.cn (R.H.); xiang.ma@zafu.edu.cn (X.M.); jiangnengjing@163.com (N.J.); zhouwuduo@njau.edu.cn (W.Z.); GC1594140795@126.com (C.G.); zmr787635834@163.com (M.Z.); 2 Huaian Academy, Nanjing Agricultural University, Huaian 223005, China; niupeipei2@126.com (P.N.); zongpingzhang@126.com (Z.Z.); 3 Huaiyin Pig Breeding Farm of Huaian City, Huaian 223322, China; 13912078083@139.com (Q.L.); 15261762741@163.com (J.Z.)

**Keywords:** *AHR* gene, *rs339939442*, chinese pigs, western pigs, litter size, association analysis

## Abstract

**Simple Summary:**

Litter size is one of the most important economic traits in pig production. However, it is slow to improve litter size by traditional breeding methods. It has been paid much 1attention to improving pig litter performance by using marker-assisted selection and genomic selection. Our research indicated that *rs339939442* in the aryl hydrocarbon receptor (*AHR*) gene could be a marker to improve litter size in European commercial lines and Chinese Suhuai pigs containing genomic fragments of British Yorkshire pigs, and the litter size could be improved by selecting the individuals with favorable genotype (TT) of *rs339939442* for pig breeding.

**Abstract:**

Eastern and Southern Chinese pigs have been imported to Western countries to improve economic traits including fertility in Western pig breeds by intensive selecting Chinese advantage genes. It was reported that the selected Asian-derived non-synonymous mutations including *rs339939442* (G > T) in the aryl hydrocarbon receptor (*AHR*) gene could increase litter size in multiple European commercial lines. The objective of this study is to identify whether *rs339939442* in the *AHR* gene is polymorphic and has an influence on the litter size in 10 pig populations including five Chinese indigenous breeds, one cultivated breed, one lean-type breed, two North American lean-type breeds, and one European lean-type breed. We found that *rs339939442* had polymorphism in all 10 populations, whereas *rs339939442* was associated with litter size only in French Yorkshire (FRA-Y) and Chinese cultivated Suhuai (SH) pigs containing approximately 75% British Yorkshire pigs ancestry. Our results indicated that *rs339939442* in the *AHR* gene was a potential marker to improve litter size in European commercial lines and the pigs containing ancestries of European commercial lines, whereas this locus maybe not a causal mutation affecting the litter size but only in linkage disequilibrium with the causal mutation for litter size.

## 1. Introduction

As one of the most important reproductive traits, litter size is a complex quantitative trait, which is affected by many factors such as genetics, nutrition, parity, nutrition, the year and season, thus the heritability of litter size is very low, which is only about 0.1 [1]. Increasing litter size by using marker-assisted selection and genomic selection can bring huge economic benefits. Therefore, there have been many studies on candidate genes and causal mutations for litter size [2,3].

Chinese indigenous pigs are divided into six types including Jianghai pigs of East China and Southern Chinese pigs [4]. Jianghai pigs including Erhualian (EHL), Meishan (MS) and Jiaxinghei (JXH) pigs are well known for their high fecundity [5]. However, the reproductive performance of Bamaxiang (BMX) and Wuzhishan (WZS) pigs representing the Southern Chinese pigs are not prominent, which the average litter size was 10.07 and 6.58 for multiparous sows, respectively [5].

As early as the end of the seventeenth century, the United Kingdom had imported Southern Chinese pigs to improve the performance of their indigenous pigs and then cultivated the famous Yorkshire pigs. Since 1979, Jianghai pigs of East China including the MS breed were introduced in France and other Western countries for their exceptional characteristics such as prolificacy and superior meat quality [6,7]. Some advantage genomic fragments in Chinese pig breeds have been strongly selected and left as selection signals among European pig breeds [8]. Through genome-wide resequencing analysis and 60K chip sequencing, the *rs339939442* (G > T) of the aryl hydrocarbon receptor (*AHR*) gene was considered to be a Chinese high-yield fragment introduced into the European pig genome, and *rs339939442* in the *AHR* gene was considered to be a potential causal mutation affecting the litter size in European commercial lines [8]. The *AHR* gene is a ligand-activating transcription factor belonging to the PER-ARNT-SIM (PAS) family and it can mediate a variety of in vivo biological processes such as immune system balance, cell growth and proliferation, vascular tissue remodeling [9,10]. It has been found that the *AHR* gene can affect reproductive traits in mice and pigs [11,12]. However, it is still unknown that whether the *rs339939442* in the *AHR* gene is polymorphic in Eastern and Southern Chinese pigs and whether this single nucleotide polymorphism (SNP) is associated with the litter size of Chinese indigenous pigs, newly cultivated pigs, lean-type pigs and North American lean meat pigs.

In this research, a total of 10 pig populations including the Eastern Chinese Jianghai pig breeds (EHL, MS and JXH), the Southern Chinese pig breeds (BMX and WZS), Chinese cultivated Suhuai (SH) pigs, which contains approximately 25% Chinese Huai pigs and 75% British Yorkshire pigs ancestries [13], Chinese lean-type Taiwanese Landrace pigs (TPE-L), Western commercial pigs including Canadian Yorkshire (CAN-Y) pigs, American Yorkshire (USA-Y) pigs and French Yorkshire (FRA-Y) pigs were used to study whether the *rs339939442* in the *AHR* gene is polymorphic and associated with the litter size in Chinese and Western pigs.

## 2. Materials and Methods

### 2.1. Animals and Data Collection

All experimental procedures and animals were carried out according to the Guidelines for the Care and Use of Laboratory Animals prepared by the Nanjing Agricultural University Animal Care and Use Committee (Certification No.: SYXK (Su) 2017-0007). The ethics committee of Nanjing Agricultural University approved this study. A total of 6943 animals from 10 diverse pig populations including five Chinese indigenous breeds representing two ecotypes, one Chinese cultivated breed, one Chinese lean-type breed, two North American lean meat pig breeds, and one European lean meat pig breed were investigated in this research (Table S1).

We investigated the Eastern Chinese Jianghai pigs and Southern Chinese pigs since they were both exported to Western countries and were representative of the high and low yield pigs in China, respectively. We collected phenotypic data and ear tissue samples of Chinese indigenous pigs from national or provincial conservation farms in China. EHL and MS pigs were collected from three conservation farms, respectively. Of them, Erhualian pigs were collected from Changzhou Jiaoxi Cooperatives of Erhualian pigs (JX-EHL), Changshu Animal Husbandry and Veterinary Station (CS-EHL) and Sutai Enterprise Co., Ltd. (ST-EHL), respectively; MS pigs were collected from Kunshan Meishan Pig Protection Co., Ltd. (KS-MS), Sutai Enterprise Co., Ltd. (ST-MS) and Taicang Pig Farm (TC-MS), respectively. The other pigs from each Chinese indigenous breed were sampled from one conservation farm. Of them, JXH pigs were collected from Qinglian, Zhejiang Food Co., Ltd.; BMX pigs were collected from Yangzhou Yiluxian Ecological Agriculture Technology Co., Ltd. and WZS pigs were collected from National Wuzhishan pig conservation farm in Lingshan Town, Haikou.

We collected phenotypic data and ear tissue samples of Chinese cultivated pigs, Chinese and Western lean-type breed from one commercial farm. Of them, SH pigs were collected from Huai’an Huaiyin Pig Breeding Farm; TPE-L pigs were collected from Guangdong Wangjiang Pig Co., Ltd.; CAN-Y pigs were collected form Fujian Yichun Agricultural Development Co., Ltd.; USA-Y pigs were collected from Jinggangshan Aoxinhuafu Breeding Co., Ltd. and FRA-Y pigs were collected from Henan Xinda Animal Husbandry Co., Ltd. (Table S1).

During the consecutive years (2008–2018), data of total number of born (TNB) and number of born alive (NBA) were collected from 2367 parities of 574 EHL sows (1478 parities of 343 JX-EHL sows, 550 parities of 156 CS-EHL sows and 339 parities of 75 ST-EHL sows), 1708 parities of 395 MS sows (1210 parities of 263 KS-MS sows, 283 parities of 79 TC-MS sows and 215 parities of 54 ST-MS sows), 2465 parities of 845 SH sows, 10,264 parities of 3095 TPE-L sows, 3101 parities of 1252 CAN-Y sows, 704 parities of 359 FRA-Y sows and 1326 parities of 365 USA-Y pigs (Table S1). It is a pity that there is no detailed litter size record for JXH, BMX and WZS.

Ear tissue from the right ear of each sow was collected and stored in 75% alcohol for genomic DNA extraction. Since some sows were eliminated from the farm before sampling, we only collected ear tissues of 363 EHL sows including 177 JX-EHL sows, 116 CS-EHL sows and 70 ST-EHL sows; 273 MS sows including 153 KS-MS sows, 47 ST-MS sows and 73 TC-MS sows; 62 JXH sows; 32 BMX sows; 20 WZS sows; 314 SH sows; 351 TPE-L sows; 425 CAN-Y sows; 345 USA-Y sows and 359 FRA-Y sows for subsequent genomic DNA extracting and genotyping (Table S1).

### 2.2. DNA Extraction and Single Nucleotide Polymorphisms Genotyping

Genomic DNA was extracted from ear tissue using a standard phenol/chloroform method. The DNA concentration and integrity were detected to select high-quality genomic DNAs for subsequent genotyping. The primers (F: AGCAGCAGCAACAACTGTGT, R: GACACAGCTCCACCATAGCA) of the *rs339939442* were used to amplify a fragment of 427 bp [8]. PCR was carried out with an initial denaturing step at 98 °C for 2 min and then 35 cycles at 98 °C for 10 s, 61 °C for 10 s and 72 °C also for 10 s, and a final extension at 72 °C for 2 min. The PCR products are sent to the company (TsingKe, Nanjing, China) for Sanger sequencing. After the sequencing finished, DNAman software was used to verify the accuracy of the sequence, and then Chromas software was used to determine the SNP loci and for genotyped. According to the reference genome information, T is the variant (G > T).

### 2.3. Statistical Analysis

#### 2.3.1. Phenotypic and Gene Frequency Descriptive Statistics

The descriptive statistics and difference analysis of NBA and TNB for seven pig populations (EHL, MS, SH, TPE-L, CAN-Y, FRA-Y and USA-Y pigs) were performed by SPSS 20.0 software (SPSS Inc., Chicago, IL., USA). The records of TNB and NBA of less than five were removed as previously reported [14]. Since there is no genetic exchange among different farms of the EHL or MS pig breed, the phenotypic data from different herds within a pig breed cannot be combined to calculate the estimated breeding values (EBV) value of TNB and NBA. Here we only use the phenotypic data of JX-EHL pig, which contains more than 150 individuals, to calculate the EBV value of TNB and NBA of EHL pigs. Meanwhile, we used the phenotypic data of KS-MS pig to calculate the EBV value of TNB and NBA of MS pigs. Microsoft Excel 2013 was used for gene frequency analysis in 10 pig populations.

#### 2.3.2. EBV Calculation

EBV_NBA_ and EBV_TNB_ for seven pig populations (JX-EHL, KS-MS, SH, TPE-L, CAN-Y, FRA-Y and USA-Y pigs) with more than 150 individuals were determined based on the pooled data across all parties using the DMU software [15].

(1) $$Y_{\mathrm{ijklmn}}=\mu +{(H/Y/S)}_{i}+{\left(\mathrm{HYS}/\mathrm{YS}\right)}_{j}+{\mathrm{PA}}_{k}+P_{l}+A_{m}+B_{n}+e_{\mathrm{ijklmn}}$$

where Y_ijklmn_ is the litter size phenotype; μ is the overall mean; (H/Y/S)_i_ is the fixed effect of herd or year or season, (HYS/YS)_j_ is the random effect of herd-year-season or year-season: when one population has multiple herds, H_i_ as the fixed effect, HYS_j_ as the random effect [16]; when one population only has one herd, (Y/S)_i_ is the fixed effect of year or season according to the *p* of the association between the year/season and NBA and TNB using ANOVA analysis of SPSS (Table S2), YS_j_ is the random effect of year-season; PA_k_ is the fixed effect of parity; P_l_ is the random effect of permanent environment; A_m_ represents random additive genetic effect; B_n_ is the random effect of service boar and e_ijklmn_ is the random residual effect.

#### 2.3.3. Two Association Analysis Methods

Association analyses of *rs339939442* with NBA and TNB of all parity in EHL, MS, SH, TPE-L, CAN-Y, USA-Y and FRA-Y pigs were conducted. Two analytical methods were used to identify whether *rs339939442* in the *AHR* gene is a potential causal mutation affecting the litter size.

The first method of association analysis of *rs339939442* with litter size was performed by the mixed linear model with SAS 9.2 software (SAS Institute lnc, Cary, NC, USA):

(2) $$Y_{\mathrm{ijklmno}}=\mu +{(H/Y/S)}_{i}+{\left(\mathrm{HYS}/\mathrm{YS}\right)}_{j}+{\mathrm{PA}}_{k}+P_{l}+G_{m}+A_{n}+B_{o}+e_{\mathrm{ijklmno}}$$

where Y_ijklmno_ is the litter size phenotype; μ is the overall mean; (H/Y/S)_i_ is the fixed effect of herd or year or season, (HYS/YS)_j_ is the random effect of herd-year-season or year-season: when one population has multiple herds, H_i_ as the fixed effect, HYS_j_ as the random effect [16], when one population has only one herd, (Y/S)_i_ is the fixed effect of year or season according to the *p* of the association between the year or season and NBA and TNB using ANOVA analysis of SPSS software (Table S1), YS_j_ is the random effect of year-season; PA_k_ is the fixed effect of parity; Pl is the random effect of permanent environment; Gm is the fixed effect of genotype; A_n_ represents random additive genetic effect; B_o_ is the random effect of service boar and e_ijklmno_ is the random residual effect. Since the limited number of populations, we did not perform association analyses of *rs339939442* with the litter size of Southern Chinese pigs including WZS and BMX pigs.

The second method of association analysis of *rs339939442* with litter size was performed by using ANOVA analysis of SPSS software to calculate the association between *rs339939442* and EBVNBA/EBVTNB for each pig population [8]. The sample size for calculation of the EBVNBA/EBVTNB for each pig population was larger than that of association analysis since some sows had been eliminated before sampling in each pig population.

## 3. Results

### 3.1. Phenotypic Variation of NBA and TNB within Seven Pig Populations

The phenotype description of the NBA and TNB of the seven pig populations were shown in Table 1 and Table 2. There was no difference in litter size between EHL and MS pigs, which both were Chinese Jianghai pigs. The litter size of EHL and MS pigs were significantly higher than that of SH (Chinese cultivated pigs) and TPE-L (Chinese lean meat pigs) pigs (*p* < 0.01). In Western lean meat pig populations, CAN-Y sows have the highest litter size, while USA-Y sows have the lowest reproductive performance (*p* < 0.01). We noticed that the variation coefficients of NBA and TNB exceed 20% in all pig populations.

### 3.2. Genetic Polymorphism in Ten Pig Populations

The *rs339939442* was identified to be polymorphic in all 10 pig populations (EHL, MS, BMX, WZS, JXH, SH, TPE-L, CAN-Y, USA-Y and FRA-Y) and the data of gene polymorphism were shown in Table 3. The genotypes of this locus represented skewed distribution in most populations including EHL, MS, BMX, WZS, JXH, SH and USA-Y. There were no TT genotype individuals in JXH, BMX and WZS pig populations. Except for TPE-L pigs, the frequency of TT genotype was the least in all three genotypes within the other nine pig populations.

### 3.3. Association Analysis of rs339939442 in the AHR Gene with Litter Size

#### 3.3.1. Association Analysis of *rs339939442* in the *AHR* Gene with NBA/TNB

The results of association of *rs339939442* in the *AHR* gene with the NBA/TNB in EHL (JX-EHL, CS-EHL and ST-EHL), MS (KS-MS, ST-MS and TC-MS), SH, TPE-L, CAN-Y, USA-Y and FRA-Y pigs were shown in Table 4. In SH pig population, *rs339939442* was significantly associated with NBA/TNB (*p* < 0.05), and the TT genotype was the advantageous genotype. In FRA-Y population, there was a tendency (*p* < 0.1) of association between *rs339939442* with litter size. Whereas, there were no association between *rs339939442* in the *AHR* gene and the litter size of EHL, MS, TPE-L, CAN-Y and USA-Y pigs.

#### 3.3.2. Association Analysis between *rs339939442* in the *AHR* Gene and EBV_NBA_/EBV_TNB_

The result of the association of *rs339939442* in the *AHR* gene with the EBV_TNB_ and EBV_NBA_ in seven pig populations (JX-EHL, KS-MS, SH, TPE-L, CAN-Y, USA-Y and FRA-Y) is shown in Table 5. The *rs339939442* was significantly associated with the EBV_NBA_ and EBV_TNB_ (*p* < 0.01) in the SH population. In the FRA-Y population, the *rs339939442* was also significantly associated with EBV_NBA_ and EBV_TNB_ (*p* < 0.01). Whereas, there were no association between *rs339939442* in the *AHR* gene and litter size in the other five pig populations.

## 4. Discussion

The litter size trait is the main focus of the swine industry and is affected by many environmental and genetic factors [17,18,19]. Detection of the exact genetic markers or genes associated with litter size in pigs may have a significant impact on improving reproductive traits and thus improve the accuracy of selection [20].

*AHR* gene is expressed in the liver, kidney, lung, thymus, brain and reproductive organs of many mammals [21,22,23]. It is a gene found in recent years that may affect the growth of follicles by regulating estradiol secretion [24]. It also was found to play an important role in regulating the number of sinusoidal follicles and the formation of primary follicles [25]. The activated expression of the *AHR* gene is susceptible to tetrachlorodibenzo-p-dioxin (TCDD). In boars, the combination of *AHR* and TCDD can cause endocrine disorders, reduced sperm, altered sexual behavior and reduced fertility [26]. In sows, the combination of TCDD and *AHR* affects the follicular phase and luteinizing steroid hormones in the sow’s estrus cycle [27]. Activated *AHR* can cause ovarian dysfunction in females and decrease and degeneration of sperm cells in males. Histomorphological analysis was performed using ovarian tissue from 4d-born mice, and it was found that the number of starting follicles in the ovaries of *AHR* gene-deficient mice was approximately twice that of wild-type mice [28]. It was also found that the *AHR* gene was considered to be a Chinese high-yield genomic fragment introduced into the Western pig genome to improve the litter size and *rs339939442* in the *AHR* gene was considered to be a potential causal mutation for litter size in European commercial lines [8]. In order to identify whether *rs339939442* is polymorphic and has an influence on the litter size in Chinese and Western pigs, 10 diverse pig populations including five Chinese indigenous breeds, one Chinese cultivated breed, one Chinese lean-type breed, two North American lean meat pig breeds and one European lean meat pig breed were chosen to carry out the test.

NBA and TNB were chosen as the phenotypes of this research, and many factors that affect them such as herd, year, season and boar had been considered in association analysis. EBV for TNB and NBA of pig were thus explored to avoid systematic environmental impacts on our conclusion drawn in this study [8]. The sample size for descriptive analysis of phenotype and the calculation of the EBV_NBA_/EBV_TNB_ was larger than that of association analysis since some sows had been eliminated before sampling. Moreover, the sample size for Chinese indigenous pigs including EHL and MS pig is somewhat difficult to increase any more [14].

EHL pig is the representative of the high fecundity of Chinese local pigs [29], and MS pig is one of the Chinese breeds that were introduced into France in 1979 due to high fertility [30]. We found that the litter size of EHL and MS pigs were significantly higher than that of SH and TPE-L pigs. SH pig population is a unique cultivar possessing ancestries of Chinese Huai pig and British Yorkshire pig [13]. There are only a few related studies on reproductive traits for SH pig breed [31,32,33]. Descriptive statistics of NBA and TNB indicated that the litter size was low in SH pigs, and it is urgent to improve their litter size through molecular breeding. The strictly organized breeding program was adopted to improve and develop livestock breeds, Britain, in particular, was the main center of the early improvement of pig breeds and cultivated the famous Yorkshire pigs [34]. FRA-Y was regarded as the pigs most likely to contain ancestries of Chinese local pigs [35]. Canada and the United States are far from the origin country of the Yorkshire-Britain and FRA-Y, CAN-Y and USA-Y were thus used in the study. Coefficient of variation (CV) of TNB and NBA reached more than 20% in the seven studied populations including EHL, MS, SH, TPE-L, CAN-Y, USA-Y and FRA-Y pigs, indicated a large phenotypic variation in litter size within each population.

By sequencing the PCR amplification products, *rs339939442* locus was found located in the 10th exon region of the *AHR* gene, and the *rs339939442* was identified to be polymorphic in all 10 pig populations. Moreover, the genotype frequency of *rs339939442* represented skewed distribution in most populations including EHL, MS, BMX, WZS, JXH, SH and USA-Y. The frequency of the TT genotype was small and was not more than 0.2 in Chinese indigenous pig groups, and even no TT genotype individuals were found in the BMA, WZS and JXH pig populations. The small size of the population might be the main cause for the absence of the TT genotype in these three pig populations, in which the number was only 32, 20 and 62 respectively. Interestingly, the frequency of the TT genotype was small and was not more than 0.2 in all 10 pig groups but Taiwanese Landrace pigs (TPE-L). This result is maybe due to: (1) the different genetic background between TPE-L pigs and the other populations; (2) since the *AHR* gene is involved in multiple traits [8,36], it could be that different direct or indirect selection acted on the alleles of *rs339939442* under different breeding goals between TPE-L pigs and the other populations.

We found that *rs339939442* in the *AHR* gene was associated with the NBA and TNB of SH pig population through two methods. This may be attributable to that SH pigs contained 75% British Yorkshire pigs ancestry. We also found *rs339939442* was associated with the NBA and TNB of FRA-Y. The result was consistent with that *rs339939442* locus was associated with the litter size of European pigs [8]. Previous research has also shown that the *AHR* gene influence estrous cycle and ovarian development and fertility [12,25,37]. Therefore, our results and previous results can suggest that the *AHR* gene has a certain role in the reproduction process. We found that TT genotype was the advantageous genotype for NBA and TNB. This result indicated that it is an effective way to select TT genotyped individuals to increase the litter size in SH and FRA-Y pigs. Our results verified that *rs339939442* in the *AHR* gene was a potential marker to improve litter size in European commercial lines and the pigs containing ancestries of European commercial lines.

However, there were no association between *rs339939442* in the *AHR* gene and the litter size of EHL, MS, TPE-L, CAN-Y and USA-Y pigs. Association of *rs339939442* in the *AHR* gene with litter size is inconsistent among different pig populations like other candidate gene markers for litter size [38]. This result is most likely due to: (1) the different genetic background between European commercial lines and these populations and (2) *rs339939442* is only in linkage disequilibrium with the causal mutation of litter size. Since recombination exists between the SNP and the causal mutation in the EHL, MS, TPE-L, CAN-Y and USA-Y pigs, no significant association can be detected.

## 5. Conclusions

Taken together, *rs339939442* in the *AHR* gene had polymorphism in both Chinese indigenous pigs and Western commercial pigs, whereas *rs339939442* was significantly associated with litter size only in FRA-Y and Chinese cultivated SH pigs containing British Yorkshire pigs ancestry. Our results indicated that *rs339939442* was a potential marker to improve litter size in European commercial lines and the pigs containing ancestries of European commercial lines and *rs339939442* maybe only in linkage disequilibrium with the causative mutation for litter size.

## Figures and Tables

**Table 1:** Descriptive statistics of the number of born alive (NBA) in seven pig populations.

Category	Breed	N_sow_	N_parity_	Range ^1^	Mean ± SE	CV%
Chinese indigenous pigs	EHL	571	2343	6–25	13.30 ± 0.06	23.05
	MS	395	1684	6–23	13.11 ± 0.07	20.32
Chinese cultivated breed	SH	835	2366	6–17	9.88 ± 0.04	20.75
Chinese lean meat pigs	TPE-L	3023	9903	6–21	10.58 ± 0.03	23.75
Western lean meat pigs	CAN-Y	1235	2992	6–26	14.23 ± 0.06	23.06
	USA-Y	365	1215	6–18	10.31 ± 0.07	23.25
	FRA-Y	359	697	6–19	11.94 ± 0.10	21.99

N_sow_ represents the number of sows; N_parity_ represents the number for total parities; CV represents the coefficient of variation; EHL represents Erhualian pig; MS represents Meishan pig; SH representative Suhuai pig; TPE-L represents Chinese lean-type Taiwanese Landrace pig; CAN-Y represents Canadian Yorkshire pig; USA-Y represents American Yorkshire pig; FRA-Y presents French Yorkshire pig. ^1^ Range of NBA from the smallest value to the largest value.

**Table 2:** Descriptive statistics of the total number of born (TNB) in seven pig populations.

Category	Breed	N_sow_	N_parity_	Range ^1^	Mean ± SE	CV%
Chinese indigenous pigs	EHL	574	2367	6–29	13.90 ± 0.07	23.81
	MS	396	1708	6–24	13.83 ± 0.07	21.14
Chinese Cultivated breed	SH	845	2465	6–20	10.76 ± 0.05	20.62
Chinese lean meat pigs	TPE-L	3095	10,264	6–26	11.20 ± 0.03	24.26
Western lean meat pigs	CAN-Y	1252	3101	6–28	15.76 ± 0.06	22.50
	USA-Y	365	1236	6–19	10.78 ± 0.07	23.48
	FRA-Y	359	704	6–20	12.26 ± 0.10	20.60

N_sow_ represents the number of sows; N_parity_ represents the number for total parities; CV represents the Coefficient of variation; EHL represents Erhualian pig; MS represents Meishan pig; SH representative Suhuai pig; TPE-L represents Chinese lean-type Taiwanese Landrace pig; CAN-Y represents Canadian Yorkshire pig; USA-Y represents American Yorkshire pig; FRA-Y presents French Yorkshire pig. ^1^ Range of TNB from the smallest value to the largest value.

**Table 3:** Genetic polymorphism analysis in 10 pig populations.

Category	Breed	N	Genotype Frequency			Allele Frequency	
			GG (n)	GT (n)	TT (n)	G	T
Chinese Jianghai pigs	EHL	363	0.791 (287)	0.198 (72)	0.011 (4)	0.890	0.110
	MS	273	0.740 (202)	0.245 (67)	0.015 (4)	0.863	0.137
	BMX	32	0.875 (28)	0.125 (4)	0 (0)	0.938	0.062
South China pigs	WZS	20	0.950 (19)	0.050 (1)	0 (0)	0.975	0.025
	JXH	62	0.984 (61)	0.016 (1)	0 (0)	0.992	0.008
Chinese cultivated breed	SH	314	0.634 (199)	0.334 (105)	0.032 (10)	0.801	0.199
Chinese lean meat pigs	TPE-L	351	0.142 (50)	0.490 (172)	0.368 (129)	0.387	0.613
Western lean meat pigs	CAN-Y	425	0.301 (128)	0.525 (223)	0.174 (74)	0.564	0.436
	USA-Y	345	0.751 (259)	0.229 (79)	0.020 (7)	0.865	0.135
	FRA-Y	359	0.418 (150)	0.485 (174)	0.097 (35)	0.660	0.340

N represents the total genotyped number; n represents the number of different genotyped individuals; EHL represents Erhualian pig; MS represents Meishan pig; BMX represents Bamaxiang pig; WZS represents Wuzhishan pig; JXH represents JiaXinghei pig; SH representative Suhuai pig; TPE-L represents Chinese lean-type Taiwanese Landrace pig; CAN-Y represents Canadian Yorkshire; USA-Y represents American Yorkshire; FRA-Y presents French Yorkshire.

**Table 4:** Association of *AHR* gene with the number of born alive (NBA) and the total number of born (TNB) in seven pig populations.

Breed	Genotype	N	NBA		TNB	
			Mean ± SE	*p*	Mean ± SE	*p*
EHL	GG	287	13.63 ± 0.17	0.359	14.55 ± 0.20	0.613
	GT	72	13.70 ± 0.24		14.69 ± 0.27	
	TT	4	14.71 ± 0.76		15.24 ± 0.81	
MS	GG	202	12.78 ± 0.10	0.419	13.62 ± 0.16	0.308
	GT	67	13.04 ± 0.18		13.96 ± 0.23	
	TT	4	13.06 ± 0.75		13.90 ± 0.84	
SH	GG	199	9.63 ± 0.17 ^b^	0.003	10.69 ± 0.20 ^b^	0.004
	GT	105	9.97 ± 0.20 ^a,b^		11.14 ± 0.22 ^a^	
	TT	10	10.82 ± 0.42 ^a^		11.66 ± 0.45 ^a,b^	
TPE-L	GG	50	11.15 ± 0.32	0.968	11.51 ± 0.33	0.726
	GT	172	11.15 ± 0.24		11.72 ± 0.25	
	TT	129	11.10 ± 0.26		11.75 ± 0.26	
CAN-Y	GG	128	14.68 ± 0.27	0.558	16.62 ± 0.31	0.654
	GT	223	14.91 ± 0.23		16.86 ± 0.26	
	TT	74	14.98 ± 0.31		16.82 ± 0.34	
USA-Y	GG	259	10.23 ± 0.10	0.595	10.69 ± 0.14	0.501
	GT	79	10.37 ± 0.16		10.90 ± 0.20	
	TT	7	9.90 ± 0.53		10.36 ± 0.60	
FRA-Y	GG	150	11.66 ± 0.29	0.076	12.03 ± 0.24	0.073
	GT	174	12.13 ± 0.29		12.49 ± 0.23	
	TT	35	12.14 ± 0.42		12.49 ± 0.37	

N represents the genotyped number; ^a, b^ values within the column indicated by different superscripts are significantly (*p* < 0.05) different; EHL represents Erhualian pig; MS represents Meishan pig; SH representative Suhuai pig; TPE-L represents Chinese lean-type Taiwanese Landrace pig; CAN-Y represents Canadian Yorkshire; USA-Y represents American Yorkshire; FRA-Y presents French Yorkshire.

**Table 5:** Association of *AHR* gene with the EBV_NBA_ and EBV_TNB_ in seven pig populations.

Breed	Genotype	N	NBA		TNB	
			Mean ± SE	*p*	Mean ± SE	*p*
JX-EHL	GG	122	−0.337 ± 0.060	0.176	−0.327 ± 0.057	0.222
	GT	47	−0.124 ± 0.092		−0.134 ± 0.091	
	TT	3	−0.084 ± 1.068		−0.221 ± 0.912	
KS-MS	GG	99	0.021 ± 0.028	0.398	0.019 ± 0.037	0.189
	GT	51	0.069 ± 0.047		0.132 ± 0.065	
	TT	3	0.205 ± 0.169		0.246 ± 0.190	
SH	GG	199	−0.014 ± 0.031 ^B,b^	0.001	−0.012 ± 0.017 ^b^	0.004
	GT	105	0.097 ± 0.047 ^A,B,a^		0.062 ± 0.029 ^a,b^	
	TT	10	0.492 ± 0.218 ^A,a^		0.212 ± 0.108 ^a^	
TPE-L	GG	50	0.318 ± 0.077	0.533	0.169 ± 0.085	0.860
	GT	172	0.320 ± 0.044		0.213 ± 0.046	
	TT	129	0.247 ± 0.05		0.181 ± 0.057	
CAN-Y	GG	128	0.348 ± 0.046	0.311	0.405 ± 0.045	0.188
	GT	223	0.388 ± 0.040		0.431 ± 0.040	
	TT	74	0.476 ± 0.072		0.555 ± 0.077	
USA-Y	GG	259	−0.072 ± 0.020	0.650	−0.052 ± 0.021	0.527
	GT	79	−0.035 ± 0.034		−0.005 ± 0.036	
	TT	7	−0.027 ± 0.179		−0.008 ± 0.184	
FRA-Y	GG	150	−0.008 ± 0.004 ^B,b^	0.003	−0.021 ± 0.013 ^B,b^	0.006
	GT	174	0.008 ± 0.003 ^A,a^		0.030 ± 0.011 ^A,a^	
	TT	35	0.007 ± 0.007 ^A,B,a,b^		0.032 ± 0.022 ^A,B,a,b^	

N represents the genotyped number; ^A, B^ values within the column indicated by different superscripts are significantly (*p* < 0.01) different; ^a, b^ values within the column indicated by different 1superscripts are significantly (*p* < 0.05) different; JX-EHL represents Jiaoxi Erhualian pig; KS-MS represents Kunshan Meishan pig; SH representative Suhuai pig; TPE-L represents Chinese lean-type Taiwanese Landrace pig; CAN-Y represents Canadian Yorkshire; USA-Y represents American Yorkshire; FRA-Y presents French Yorkshire.

## Supplementary Materials

The following are available online at https://www.mdpi.com/2076-2615/10/1/11/s1, Table S1: The specific composition for different analysis of the animals in this study, Table S2: Correlation analysis between year/season and litter size.
