# Supplementary material for: Association of Rs339939442 in the AHR Gene with Litter Size are Inconsistent among Chinese Indigenous Pigs and Western Commercial Pigs
Source: Animals (Basel). 2019 Dec 19;10(1):11. doi: 10.3390/ani10010011 (PMC7023200; doi:10.3390/ani10010011)
Supplement: Supplementary file 1 [file animals-10-00011-s001.pdf]

Supplementary table

Table S1. The specific composition for different analysis of the animals in this study.

Category	Breed	$n_{description}$	$n_{litter1}$	n_{EBV}	$n_{litter2}$	$n_{genotype}$	$n_{litter3}$
Chinese Jianghai pigs	JX-EHL	343	1478	343	1478	177	827
	CS-EHL	156	550	/	/	116	497
	ST-EHL	75	339	/	/	70	314
	KS-MS	263	1210	263	1210	153	857
	ST-MS	54	215	/	/	47	201
	TC-MS	79	283	/	/	73	266
Southern Chinese pigs	JXH	/	/	/	/	62	/
	BMX	/	/	/	/	32	/
	WZS	/	/	/	/	20	/
Chinese cultivated breed	SH	845	2465	845	2465	314	1242
Chinese lean meat pigs	TPE-L	3095	10264	3095	10264	351	741
Western lean meat pigs	CAN-Y	1252	3101	1252	3101	425	1165
	USA-Y	365	1326	365	1326	345	1150
	FRA-Y	359	704	359	704	359	697

$n_{description}$, n_{EBV} and $n_{genotype}$ respectively represents the number of individuals for descriptive analysis, EBV calculation and genotyping. $n_{litter1}$, $n_{litter2}$ and $n_{litter3}$ respectively represents the number of litters used for descriptive analysis, EBV calculation and genotyping. JX-EHL represents Jiaoxi Erhualian pig; CS-EHL represents Changshu Erhualian pig; ST-EHL represents Sutai Erhualian pig; KS-MS represents Kunshan Meishan pig; ST-MS represents Sutai Meishan pig; TC-MS represents Taicang Meishan pig; JXH represents JiaXinghei pig; BMX represents Bamaxiang pig; WZS represents Wuzhishan pig; SH representative Suhuai pig; TPE-L represents Taiwanese Landrace pig; CAN-Y represents Canadian Yorkshire pig; USA-Y represents American Yorkshire pig; FRA-Y presents French Yorkshire pig.

Table S2. Correlation analysis between year/season and litter size.

**Breed**	**NBA**		**TNB**	
	p_{year}	p_{season}	p_{year}	p_{season}
KS-MS	0.000	0.801	6.00E-06	0.694
SH	0.000	0.044	1.52E-04	0.045
CAN-Y	0.000	0.000	4.00E-06	0.045
USA-Y	0.135	0.098	0.400	0.182
FRA-Y	1.41E-04	3.80E-05	5.30E-05	9.30E-05

p_{year} and p_{season} respectively represents the p value of the correlation between the year and NBA/TNB and the correlation between the season and NBA/TNB. KS-MS represents Kunshan Meishan pig; SH representative Suhuai pig; CAN-Y represents Canadian Yorkshire pig; USA-Y represents American Yorkshire pig; FRA-Y presents French Yorkshire pig.
